# Angled Insertion of Microneedles for Targeted Antigen Delivery to the Epidermis

**DOI:** 10.3390/pharmaceutics14020347

**Published:** 2022-02-01

**Authors:** Rohan Murty, Abishek Sankaranarayanan, Isabella I. Bowland, Juan Mena-Lapaix, Mark R. Prausnitz

**Affiliations:** School of Chemical and Biomolecular Engineering, Georgia Institute of Technology, Atlanta, GA 30332, USA; rmurty6@gatech.edu (R.M.); asankara6@gatech.edu (A.S.); ibowland3@gatech.edu (I.I.B.); menalapa@gatech.edu (J.M.-L.)

**Keywords:** coated microneedle patch, targeted drug delivery, skin epidermis, allergy immunotherapy, peanut protein, optical coherence tomography

## Abstract

Peanut and tree nut allergies account for most food-induced anaphylactic events. The standard allergy immunotherapy approach involves subcutaneous injection, which is challenging because severe adverse reactions can occur when antigens spread systemically. Allergen localization within the epidermis (i.e., the upper 20–100 µm of skin) should significantly reduce systemic uptake, because the epidermis is avascular. Microneedle (MN) patches provide a convenient method for drug delivery to the skin, but they generally target both epidermis and dermis, leading to systemic delivery. In this study, we adapted MN technology for epidermal localization by performing angled insertion of 250 µm–long MNs that limits MN insertion depth mostly to the epidermis. We designed a biplanar insertion device to aid the repeatability of angled insertions into porcine skin ex vivo at specified angles (90°, 45°, and 20°). When compared to 90° insertions, MN application at 20° decreased mean insertion depth from 265 ± 45 µm to 97 ± 15 µm. Image analysis of histological skin sections revealed that acute-angle insertion increased epidermal localization of delivery for antigen-coated MNs from 25% ± 13% to 70% ± 21%. We conclude that angled insertion of MNs can target antigen delivery to epidermis.

## 1. Introduction

Peanut allergies affect 1–2% of adults and 4–8% of children [1,2,3]. Furthermore, current evidence suggests that the prevalence of food allergies in the Western world has been increasing over the last few decades [1,4]. Peanut or tree nut ingestion/contact causes most severe food-induced reactions [3], accounting for more than 24,000 food-induced anaphylactic events in the United States annually [1].

**Figure 1:**
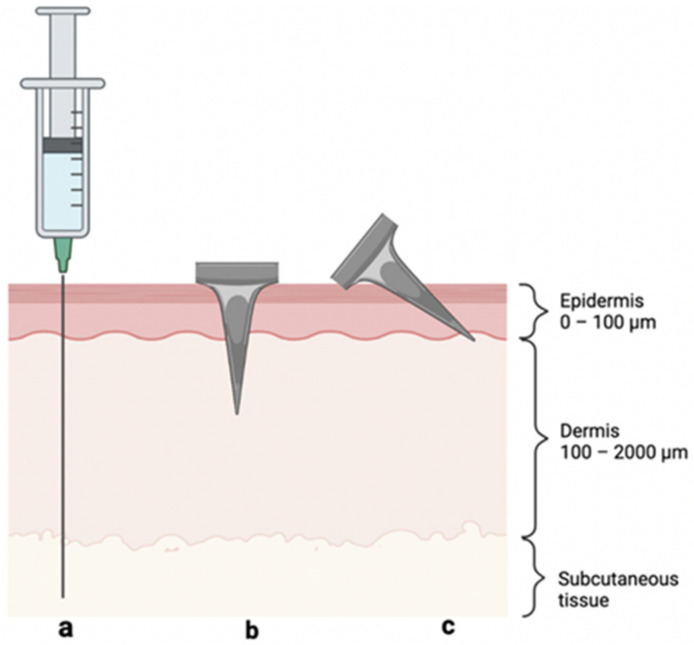
Visual representation of skin layers and three methods of antigen delivery: (**a**) hypodermic needle injection to the subcutaneous space, (**b**) perpendicular (i.e., 90°) MN insertion crossing epidermis and into the dermis, and (**c**) angled MN insertion targeting the epidermis.

Typical methods for treating peanut allergies include subcutaneous allergen immunotherapy (SCIT) [5] and oral immunotherapy (OIT) [6]. These immunotherapies are based on extended exposure of the immune system to low doses of antigen in order to induce immunological tolerance to the antigen, thereby suppressing allergic responses. SCIT is often cumbersome, as subcutaneous injection (Figure 1a) of antigens, such as *Arachis hypogaea 2*—the dominant peanut allergen [7]—can cause severe adverse effects in patients [5,8]. Additionally, young children suffer from needle phobia at a rate of up to 60–70% [9]. While OIT avoids injections, it is still in relatively early development and is hindered by patients’ increased risk of anaphylaxis and gastrointestinal discomfort, leading to withdrawal from treatment and, thus, resulting in the rapid reversal of allergen desensitization [6,10,11]. In recent years, topical immunotherapies that avoid the issues of needle phobia and gastrointestinal adverse effects have emerged (e.g., Viaskin by DBV Technologies, Bagneux, France), but they generally suffer from poor efficacy and regulatory barriers [12,13].

Adverse effects stemming from SCIT are caused by systemic uptake into the bloodstream and rapid antigen clearance by macrophages in the dermis and subcutaneous space. These processes increase the production of IgE antibodies, causing mast cells to flood the system with histamines; in extreme cases, this may result in anaphylaxis. In contrast, delivery to the avascular epidermis reduces systemic uptake, thereby increasing the relative production of IgG_4_ antibodies—i.e., those responsible for immune tolerance [14,15,16]. Consequently, both the safety and efficacy of targeted allergen immunotherapy within the skin may be improved by localizing the antigen in the epidermis, ideally without requiring a hypodermic needle, as this would significantly decrease the rate of systemic uptake, elicit the desired immune response, and address needle-phobia concerns [17] (Figure 1).

In this study, we proposed that microneedle (MN) patches may provide an effective approach of targeting antigen delivery to the epidermis. MNs are micron-scale needles that enable delivery to the skin without the need for hypodermic needle injection. Since their genesis in the 1990s, methods for fabrication, coating, and application of MNs have emerged, affording drug, vaccine, and allergen delivery [18,19,20,21,22]. For instance, vaccine-coated MN patches were successfully used to prevent the development of airway asthma in mice, suggesting promise for human translation [23]. MN patch administration of drugs for osteoporosis treatment, insulin, and inactivated influenza vaccine, among other therapeutics, has been successfully studied in human clinical trials [24,25].

MN patches assuage many of the translational barriers associated with SCIT [19]; their size/geometry provides an attractive opportunity for targeting antigen delivery to epidermal thicknesses (~100 µm) [26], and administration in the form of an MN patch bypasses the issue of needle phobia. Until now, most reports of MN patches for drug delivery involve delivery to both the epidermis and dermis (Figure 1b), and this is undesirable for allergy immunotherapy [27,28]. Limiting MN penetration depth by using MNs shorter than epidermis thickness is difficult for this application for two reasons: (i) antigen loading on such small MNs may be too low to deliver the required dose and (ii) controlled insertion of such short MNs is difficult to achieve without a specialized high-velocity insertion device [29,30].

To address these challenges, we introduce angled insertion of MNs to target antigen delivery to the epidermis (Figure 1c). In this way, we can use moderate-sized MNs (i.e., 250 µm long) that have a limited skin penetration depth due to angled insertion. Because previous epicutaneous (“on the skin”) immunotherapy (EPIT) studies have administered 50–500 µg of antigen topically [31], we decided on a maximum total target dose of 50 µg, which is probably even more than epicutaneous delivery produces in the skin, given the low expected bioavailability of topical protein delivery [32]. By assuming a patch size of 100 MNs, we defined the antigen loading target as 0.5 µg/MN, which should be suitable for inducing allergic tolerance to peanuts [15].

## 2. Materials and Methods

### 2.1. Microneedle Coating

Solid stainless-steel MN arrays were constructed by using wet-etching photo-lithographically defined needle structures from stainless-steel sheets (Tech Etch, Plymouth, MA or Moonlight Therapeutics, Atlanta, GA, USA). Each MN array consisted of five co-planar MNs, each measuring 250 μm in length. Prior to coating, MNs were treated with oxygen plasma (Plasma Cleaner PDC-32G, Harrick Plasma, Ithaca, NY, USA) to increase the steel surface’s hydrophilicity and improve coating-solution adhesion. The aqueous coating solution consisted of 2% (*w*/*v*) medium-viscosity carboxymethylcellulose sodium salt (Sigma-Aldrich, St. Louis, MO, USA) and 0.5% (*w*/*v*) Pluronic F-68 (Sigma-Aldrich). When indicated, the coating solution also contained 2% (*w*/*v*) peanut *Arachis hypogaea* (Greer Laboratories, Lenoir, NC, USA) and/or 0.2% (*w*/*v*) sulforhodamine B sodium salt (Sigma-Aldrich) as a red-fluorescent dye.

The coating solution was prepared by adding 20 mg medium viscosity CMC and 5 mg Pluronic F-68, as well as 10 mg peanut protein and/or 2 mg sulforhodamine B dye in some cases, to 1 mL deionized (DI) water; this solution was then mixed via vortexing and sonication. Next, MN arrays were mounted onto a custom-made automated dip-coating device [33] through which the MNs were dip-coated in the coating solution, using two-axis robotic control. Each MN in the array was coated 10 times, following a procedure described previously [34]. Arrays were air-dried and stored at ambient conditions (20–25 °C, 30–60% relative humidity) before insertion into skin. The amount of antigen coated onto each MN was determined by dissolving the MN coating in DI water and measuring antigen concentration by bicinchoninic acid assay [35].

### 2.2. Angled Insertion Device

To facilitate insertion of MNs into skin at a controlled angle, we developed a biplanar insertion device with an adjustable angle of elevation that was designed by using computer-aided design (CAD) software (SolidWorks, Vélizy-Villacoublay, France) and fabricated out of poly(lactic acid) (ColorFabb, Belfeld, Netherlands) by a 3D-printer (Ultimaker 3, Zaltbommel, Netherlands). The two faces of the applicator hinged around a bolt; once the desired angle was selected by rotating the angled surface, a nut was tightened around the bolt to fix the device angle in place. A single planar MN array (containing 5 MNs) was taped to the edge of a flat piece of plastic and slid down the angled face for insertion. 

We used angled wedges to control the angle of insertion of the MNs into skin during real-time imaging by optical coherence tomography (OCT). We designed these wedges by using CAD software (SolidWorks) and fabricated them by using a Form 3 printer (Formlabs, Somerville, MA, USA). The wedges were made of Grayscale resin (Formlabs). After printing, the wedges were cured in a Form Cure chamber (Formlabs). We inserted the MNs by using forceps to slide the MNs over the wedge and into the skin.

### 2.3. Insertion of Microneedles into Porcine Skin Ex Vivo

Porcine belly skin was obtained from Pel-Freez Biologicals, (Rogers, AR, USA), and the subcutaneous fat layer was removed. The skin surface was dabbed with Kim wipes (to remove excess moisture) and then fixed tautly onto a cutting board to simulate natural skin tension during MN insertion. The MNs were pressed into the skin for 30 s and then removed. Perpendicular insertions (90° angle) were performed by hand.

Angled insertions (45° and 20°) were executed by using the biplanar insertion device with an adjustable angle of elevation. To insert MNs into skin, the MN array was slid down the plane of the insertion device (fixed at the desired angle) to the skin surface, where the MNs penetrated the skin for 30 s. The MNs were then removed from the skin at the same angle.

### 2.4. Histological Analysis

Immediately after insertion, the tissue around the MN array insertion site was excised and prepared for histological sectioning by freezing in liquid nitrogen for 1–2 min in Optimal Cutting Temperature Embedding Medium for Frozen Tissue Specimens (Scigen Scientific, Gardena, CA, USA). The angle of insertion was also marked on the outside of the block for guidance during sectioning before being stored at −20 °C until sectioning. Because small-molecule dye diffusion happens on the timescale of minutes (and the large protein diffuses much slower), we did not expect significant diffusion of the antigen away from the MN insertion site.

Within minutes (or hours at most), histological analysis was conducted to assess MN insertion depth into the skin and image distribution of materials dissolved from the MN coatings in the skin. Samples were sectioned to 10–15 µm thickness by using a Leica 3050 S Cryostat (Wetzlar, Germany), with 3–5 sections being collected for each of the five insertion sites imprinted by each MN array.

Frozen sections were imaged by using a stereo microscope (Olympus SZX16, Tokyo, Japan) with red fluorescence to visualize and quantify the depth of delivery of sulforhodamine. The Autostainer XL (Leica) was used to stain sections with hematoxylin and eosin (H&E), which were then imaged by using the same microscope under bright field to determine the extent of epidermal localization.

### 2.5. Optical Coherence Tomography

MNs inserted into skin were imaged in real time by using OCT (VivoSight Dx, Michelson Diagnostics, Kent, UK) with infrared light (1305 nm) to visualize to skin depths up to 500 µm from the skin surface. The OCT scan was configured to produce 500 slices of a 6 mm × 6 mm section of the skin.

### 2.6. Statistical Analysis

Statistical analysis was performed by using OriginPro 2021b software (Northampton, MA, USA). One-way ANOVA tests were used to evaluate statistical differences between the groups for insertion depth, percent epidermal targeting, and percent peanut antigen localization in the epidermis. Significance was considered for *p* < 0.05.

## 3. Results

### 3.1. Coating Microneedles with Peanut Protein Antigen

The eventual intended application for angled MN insertions in this work is to induce tolerance to peanut and other food allergens. Consequently, we first developed a formulation and method for coating a suitable dose of peanut antigen on MN arrays. Four factors were varied to meet the target antigen loading of 0.5 µg per MN: concentration of peanut protein and CMC in the coating solution, MN length, and number of dip-coating cycles.

**Figure 2:**
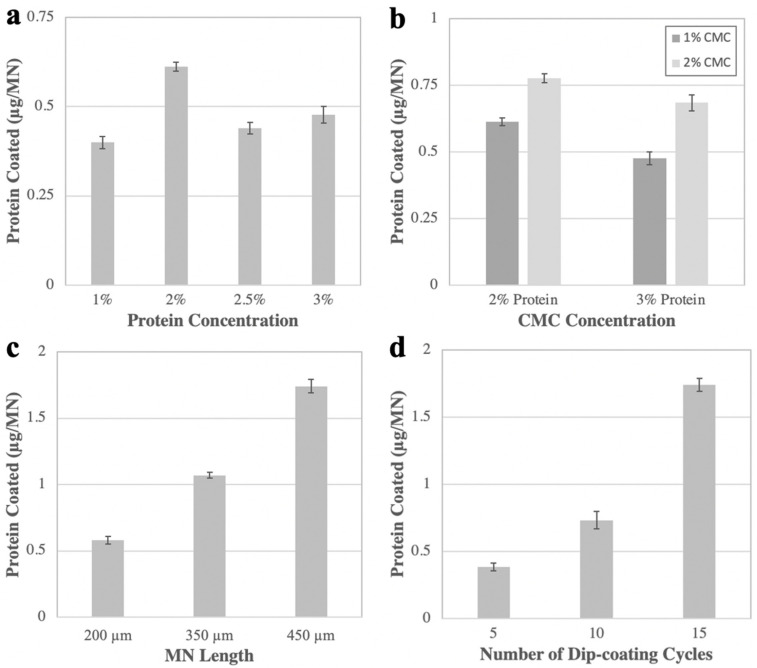
Optimization of MN coating with peanut protein antigen. The mass of protein coated is shown as a function of (**a**) protein concentration, (**b**) CMC concentration (varying from 1% to 2%, (**c**) MN length, and (**d**) number of dip-coating cycles. Unless otherwise specified, 450 µm–long MNs underwent 15 coating cycles in a solution of 2% protein, 2% CMC, 0.5% Pluronic acid *w*/*v*. Graphs show mean and standard deviation for 6 replicates.

The aqueous coating solution consisted of the peanut protein, CMC, and Pluronic F-68. While the Pluronic concentration was kept constant at 0.5% (*w*/*v*), the antigen concentration was varied from 1% to 3% (*w*/*v*), and the CMC concentration from 1% to 2% (*w*/*v*). Increasing protein concentration from 1% to 2% nearly doubled the amount of antigen loading (to ~0.6 µg/MN), but higher concentrations did not significantly improve the loading (Figure 2a). We did not pursue protein concentrations above ~2%, because they formed a highly viscous, hard-to-mix coating solution that yielded unreliable coating results (data not shown). For CMC, a 2% (*w*/*v*) concentration afforded a greater antigen loading compared to 1% (*w*/*v*), probably because the increased coating solution viscosity kept more coating solution on the MN surface during drying (Figure 2b).

We found that varying MN length from 200 to 450 µm caused a 250% increase in total antigen coating (Figure 2c). Because longer MNs could be coated with more antigen and 200 µm–long MNs met the loading target of 0.5 µg/MN, we concluded that 250 µm–long MNs would also achieve sufficient loading, as well as expected skin insertion.

We also found that increasing the number of dip-coating cycles from 5 to 15 caused a >200% increase in antigen loading (Figure 2d). However, we noted that adding more dip-coating cycles increased the likelihood of antigen coating below the base of the MN (Supplementary Materials Figure S1). This is not desirable, because any coating not on the MNs themselves will not enter the skin upon insertion. As such, we employed 10 coating cycles, since this resulted in sufficient antigen loading, while mitigating the frequency of coating past the base.

Our optimized coating method employed a formulation containing 2% (*w*/*v*) CMC, 0.5% (*w*/*v*) Pluronic F-68, 1% (*w*/*v*) peanut antigen, and 0.2% (*w*/*v*) sulforhodamine B (to assist coating visualization) that was coated onto 250 µm–long stainless-steel MNs, using 10 dip-coating cycles. Because the target dose was achieved with 1% peanut antigen, we used that concentration instead of higher concentrations; however, as long as changes to the formulation do not hinder the solution’s ability to coat uniformly on MN tips (e.g., by becoming too viscous), varying either the CMC or protein amount should not affect epidermal targeting. Although 250 µm MNs were not specifically studied in these coating experiments, we determined that 200 µm MNs were long enough to achieve the target loading, so any MN equal or greater in length would be sufficient from a dosing perspective. When considering insertion, however, 250 µm MNs were superior, as we found that reliable insertion of 200 µm (or shorter) MNs was difficult to achieve. Thus, our final MN array was selected to be long enough to achieve the target dose and insert reliably at shallow angles, while being short enough not to penetrate too far into the dermis.

While polymeric MNs were considered for this study’s immunotherapy applications (as they may have enabled greater antigen loading), we ultimately decided against this approach, as higher loading was not necessary past the 0.5 µg/MN target. Furthermore, polymeric MN patches may lack the rigidity required to insert consistently at a shallow angle, which was facilitated by the strength/sharpness of metal MNs.

### 3.2. Device for Angled Microneedle Insertion into Skin

**Figure 3:**
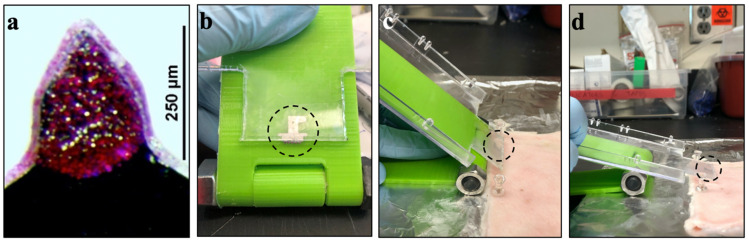
Device for angled MN insertion. (**a**) Representative MN coated with peanut protein and sulforhodamine B dye to facilitate imaging; (**b**) biplanar insertion device with MN array (circled) attached to solid surface that slides at a predetermined angle; and (**c**,**d**) device used to insert MN array into pig skin ex vivo at 45° and 20°, respectively.

Our hypothesis is that angled insertion of MNs into the skin enables more superficial delivery that targets antigen deposition in the epidermis compared to perpendicular MN insertion. While perpendicular insertion of MNs into tissue can be performed reliably by hand, angled MN insertions are more difficult to control. We therefore designed and built a biplanar insertion device that enabled control over MN insertion angle into the skin (Figure 3).

The insertion device system had three components: the device itself, a plastic substrate, and a single MN array. First, we 3D-printed a biplanar device (i.e., two sheets of plastic connected by a hinge) that could be fixed at a specified angle by tightening a nut and screw at the rotating axis (Figure 3b). We then mounted a MN array onto the plastic substrate that could slide on the surface of the insertion device, thereby bringing the MN array to the skin surface at the predefined angle (Figure 3c,d). Although only three insertion angles are reported in this work, this device afforded angular control from 90° to 5°, with respect to the tissue surface.

### 3.3. Microneedle Insertion Depth into Skin after Angled Insertion

To limit MN insertion depth primarily to the epidermis (20–100 µm thick in humans [26], we combined two strategies. First, we used short MNs of 250 µm length. We did not use MNs shorter than 250 µm, because we found that shorter MNs did not reliably insert into skin, but often just deformed the skin surface without penetration (data not shown). Second, we inserted the MNs at an angle. In this study, we used angles of 90°, 45°, and 20°. By geometry, these MNs are expected to insert 250 µm, 177 µm, and 85 µm into the skin (i.e., insertion depth (D) equals MN length (L) times the sin of the insertion angle (θ), D = L sin θ).

**Figure 4:**
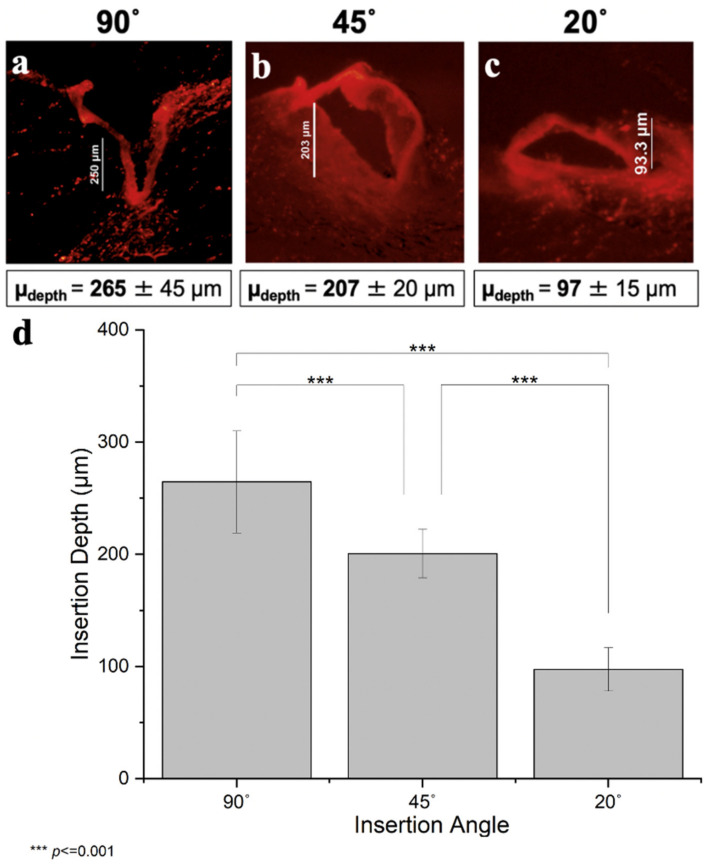
Microneedle insertion depth into skin after angled insertion. MNs measuring 250 µm in length were inserted at various angles into porcine skin ex vivo. Representative images of histological skin sections after insertion of MNs coated with red-fluorescent sulforhodamine dye at angles of (**a**) 90°, (**b**) 45°, and (**c**) 20°. Insertion depth was determined by measuring the distance from the skin surface to the tip of MN penetration into skin at the greatest depth seen in serial sections at each insertion site. (**d**) Mean and standard deviation of insertion depth after angled MN insertion for 9 replicates.

Histological analysis of MN insertions showed that MN insertion depth decreased with decreasing insertion angle (one-way ANOVA, *p* < 0.001) (Figure 4). As expected for perpendicular insertion, the mean depth was 265 ± 45 µm, which is not significantly different from the MN length of 250 µm (within 95% confidence interval of 230–300 µm, Figure 4a). After perpendicular insertion, deformation of the skin surface was evident, with the MN insertion site located at the base of a valley on the skin surface, which is consistent with prior reports [36].

The decrease in insertion depth caused by an insertion angle of 45° yielded an average depth of 207 ± 20 µm (Figure 4b). Although this is consistent with the expectation that angled insertions reduced insertion depth, this insertion depth is not sufficient for epidermal targeting. Moreover, the reduction in insertion depth was not as great as that predicted by the simple geometric calculation of 177 µm (outside the 95% confidence interval of 184–217 µm). It is not clear why this calculated value was not predictive, but it probably has to do with the non-rigid nature of skin tissue that does not conform to the shape of a perfect right triangle.

Further decreasing the insertion angle to 20° lowered insertion depth to 97 ± 15 µm (Figure 4c), which is in the range of human epidermal thickness (~100 µm), thus suggesting a viable way to increase the extent of epidermal targeting with MNs. In addition, this insertion depth is not significantly different from the calculated depth of 85 µm (within 95% confidence interval of 83–112 µm). Fluorescence images of all nine replicates for each of the three angles reported can be found in Supplementary Materials Figures S2–S4.

We also considered the degree of variability of insertion depth and found that the coefficient of variation was ~10% after 90° and 45° insertion and was ~15% after 20° insertion (Figure 4d). Sources of variability may be due to MNs sometimes “jumping” across the tissue or sometimes bending due to imperfect rigidity of the MNs, causing less uniform delivery. We noted that these effects were more prevalent during insertion at very acute angles, and this may explain the greater variability after 20° insertion. Other sources of variability include irregularities in the skin, pushing MNs too hard during insertion, performing perpendicular insertions by hand, and imperfect estimation of insertion depths from histological images. Nonetheless, variation was relatively small and suggests the ability to achieve targeted depth of insertion with good reliability.

### 3.4. Efficiency of Epidermal Targeting by Angled Microneedle Insertion

Although controlling MN insertion depth was this study’s primary goal, the scope of this work also included assessing the degree of epidermal targeting. We did this by staining the skin with H&E to identify the epidermis as a dark purple layer, while staining the dermis a pink/salmon color. In this way, the degree of epidermal localization was determined as the insertion site’s interfacial contact length with the purple-stained regions divided by the total perimeter of the insertion site within the tissue (Supplemental Figure S5). While direct measurement of epidermal delivery would be ideal, this necessitates separating the stratum corneum, epidermis, and dermis layers through interventions, such as incubating skin with enzymes or heating skin to 50–60 °C. This could introduce artefacts, such as increased dye diffusion, likely inefficient dye/protein extraction efficiency, and mechanical disruption of the tissue. Consequently, we selected an imaging-based method that provides a reasonable estimate of the degree of epidermal targeting instead.

**Figure 5:**
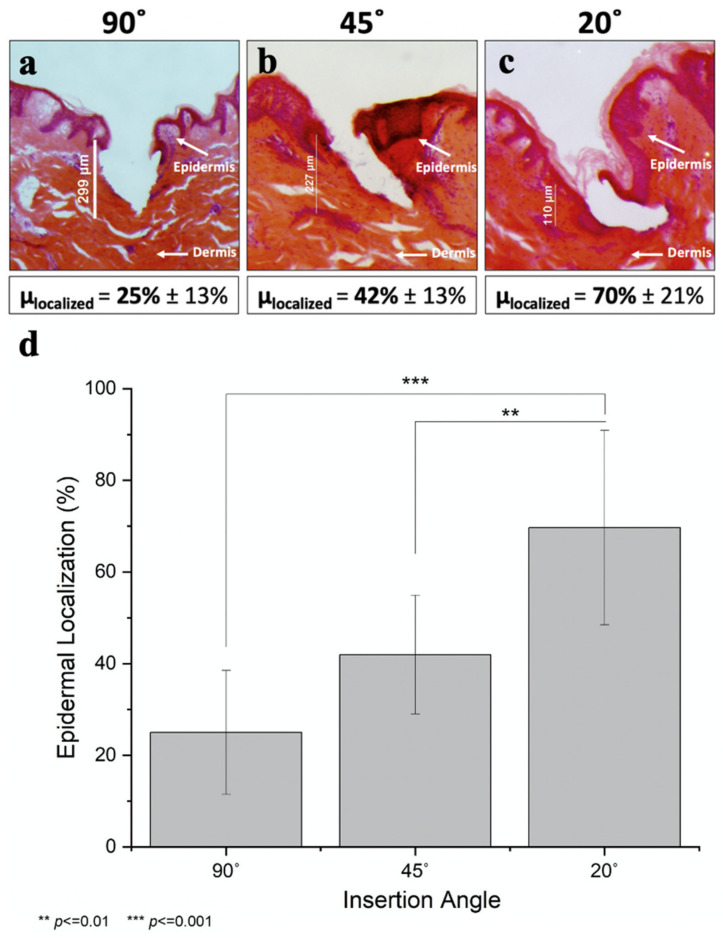
Percent epidermal localization in skin after angled insertion. MNs measuring 250 µm in length were inserted at various angles into porcine skin ex vivo. Representative H&E-stained images of histological skin sections after insertion of MNs coated with peanut antigen at angles of (**a**) 90°, (**b**) 45°, and (**c**) 20°. Percent localization was determined by dividing the insertion site’s interfacial contact length with the purple-stained epidermis regions by the total perimeter of the insertion site within the tissue. (**d**) Mean and standard deviation of percent localization after angled MN insertion for 9 replicates.

We found that decreasing the insertion angle significantly increased epidermal targeting (one-way ANOVA, *p* < 0.001; see Figure 5 and Supplementary Materials Table S1). Perpendicular insertion only afforded 25% ± 13% localization in the epidermis (Figure 5a), whereas reducing the insertion angle to 45° improved this localization nearly twofold (42% ± 13%, Figure 5b). Acute angle insertion at 20° further increased epidermal targeting to 70% ± 21% (Figure 5c). H&E-stained images of all nine replicates for each of the three angles reported can be found in Supplementary Materials Figures S6–S8.

The coefficient of variation was highest for the perpendicularly inserted MNs; this can be explained by the natural morphology of the porcine epidermis, which is structurally similar to human epidermis [37,38]. The epidermis has an undulating finger-like structure, with epidermal thickness varying as a function of position. When MNs were inserted perpendicularly to the skin surface, they randomly localized either in one of the finger-like protrusions of the epidermis or in one of the “troughs” (Figure 5a), causing significant variability in the interfacial contact distance at each insertion site. In contrast, MNs inserted at an angle almost always pierced through several of these finger-like protrusions, thereby decreasing the variability (Figure 5b,c). Additionally, MNs were sometimes bent during insertion, and this served to increase epidermal localization because the direction in which the needles were bent resulted in shallower insertion depths (Figure 5c). Because the force was always applied from above, the MNs were consistently bent in a predictable manner, tending to be parallel to the skin’s surface, thus leading to shallower depths of insertion.

### 3.5. OCT Imaging of Angled MN Insertion into Skin

**Figure 6:**
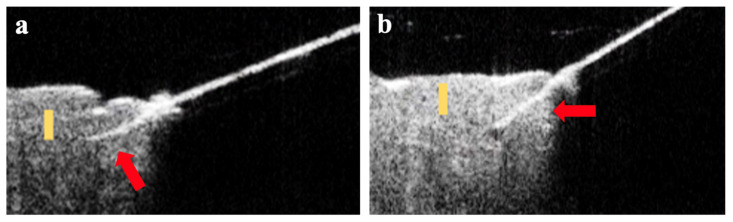
Representative OCT images of MNs inserted into skin at an angle. The MNs appear as a bright line that extends from the top right into the skin (red arrow). The light region on the lower left is skin. The dark region on the lower right is skin that is shadowed by the MN and therefore cannot be seen. The skin is oriented with the stratum-corneum-side up. (**a**) MN inserted at 20°. Yellow scale bar (100 µm) shows a MN insertion depth of approximately 100 µm. (**b**) MN inserted at 45°. Yellow scale bar (100 µm) shows a MN insertion depth of close to 200 µm.

To complement the analysis performed on histological skin sections stained after MN insertion, we also imaged the skin by OCT to visualize MNs embedded in situ in the skin (Figure 6). In these images, the MNs can be seen as a bright line extending from the upper right corner. While the skin is present across the whole bottom half of the images, it can only be seen on the left side, because the right side is shadowed from view by the angled insertion device positioned between the skin and the OCT imaging head. In these images, the MN can be seen penetrating into the skin at an angle reaching depths of approximately 100 µm after 20° insertion and approximately 200 µm after 45° insertion, which is in general agreement with the data from histological sections.

## 4. Discussion

This study introduced angled insertion of MNs into the skin to reduce insertion depth and thereby target drug delivery to the epidermis. We achieved a shallow insertion depth with increased epidermal targeting by using a short MN (250 µm long) inserted at an acute angle to the skin surface (20°) that enabled an insertion depth less than 100 µm and epidermal targeting of up to 70%.

Shallow insertion of MNs into skin has been studied before, but not via angled MN insertion. One approach involves very short MNs (e.g., <100 µm long) and their application to skin at sufficiently high velocity that the skin does not deform during the MN insertion process, thereby enabling insertion of MNs that would otherwise be too short to penetrate skin [29]. A limitation of this approach is that coating such short MNs is difficult [39] and that insertion at high velocity requires a special applicator, which adds cost, size, and complexity to the MN delivery system. Here, we distinguish between an applicator, which provides the force (e.g., using a spring) to accomplish the insertion, and our device, which is a guide that assists a manual insertion. We expect that angled MN insertion can be manually accomplished with appropriate design of a simple-to-use MN patch and its housing without the need for a bulky, expensive applicator. Another approach, using hollow MNs, involves drilling MNs into the skin by rotational insertion [40]. This minimizes skin-surface deformation and allows MN insertion to precise depths for injection into skin. This approach was used for individual MNs, because each MN needs to rotate around its own central axis, making it difficult to employ using arrays of MNs.

Many skin treatments could benefit from epidermal targeting. For example, dermatological disorders, such as basal and squamous cell carcinomas, cutaneous warts, and vitiligo, have pathology in the epidermis [41] which means that epidermal delivery can increase drug efficacy. Furthermore, because the viable epidermis (possible mediated by the basal membrane at the dermal–epidermal junction) provides a significant barrier to diffusion of macromolecules, protein delivered to epidermis may be retained there [42]. Of particular interest to this study is allergy immunotherapy, for which targeted epidermal delivery is expected to facilitate generating immune tolerance while reducing the risk of adverse side effects from systemic antigen exposure [43]. Additionally, MN-based allergy immunotherapy may reduce the need for SCIT, democratizing allergy treatment in several ways. First, immunotherapy by MN patch may substantially lower the treatment cost, as more than $4000 per year is spent on injecting medication into children suffering from food allergies [44]. Mitigating the issue of needle phobia may also increase patient compliance and expand the patient base for non-oral food-allergy immunotherapy. If MN patches for immunotherapy can be administered at home, parents may eliminate many trips to the doctor by administering patches themselves.

While this study demonstrates the feasibility of epidermal targeting by using angled MNs, additional studies are needed to fully validate the approach and assess its utility. For example, this study was limited to MN application to porcine skin ex vivo. Future studies need to assess delivery in vivo and in human skin. In addition, epidermal localization was shown mostly by histological analysis of MN tracks in the skin and deposition of coated material from the MNs. The ability of antigen or other materials delivered in this way to not only be delivered to epidermis, but remain there, even in the presence of an active vasculature, notably in the superficial dermis, needs to be studied. Drug size and other characteristics will affect local drug distribution in the skin based on rates of diffusion, tissue binding and other factors. While MNs, especially ones as short as 250 µm, have been shown to be painless and well tolerated [45,46], the safety and acceptability of angled MN insertion need to be studied. Finally, while the angled insertion device used in this study was effective, use outside the research setting would benefit from a smaller device with a fixed angle, requiring no expertise or training to use.

## 5. Conclusions

This work demonstrated that angled MN insertion can be used to target antigen delivery to the upper layers of the skin, notably the epidermis. We first developed an MN coating method to achieve a target dose of 0.5 µg peanut protein antigen (*Arachis hypogaea*) per MN by adjusting coating formulation composition, MN length, and number of dip-coating cycles. Using a custom 3D-printed biplanar angular insertion device, we inserted MNs into porcine skin ex vivo with controlled insertion angles of 20°, 45°, and 90°. Histological analysis determined that angled insertion at 20° reduced mean depth of MN penetration into skin from 265 to 97 µm compared to perpendicular (90°) insertion; this result was consistent with additional imaging performed by OCT. The extent of epidermal targeting increased from 25% after perpendicular delivery to 70% after delivery at a 20° angle. We conclude that angled MN insertion provides a promising approach to targeting drug delivery to the epidermis that may improve allergy immunotherapy and enable other applications with epidermal delivery targets.

## 6. Patents

A patent application was filed based on the work presented in this study.

## Data Availability

All data are provided in the main manuscript and Supplemental Materials.

## Supplementary Materials

The following figures and table are available online at https://www.mdpi.com/article/10.3390/pharmaceutics14020347/s1. Figure S1: MNs coated past the base. Figure S2: Fluorescent histology images of 90° MN insertion. Figure S3: Fluorescent histology images of 45° MN insertion. Figure S4: Fluorescent histology images of 20° MN insertion. Figure S5: Method for determining percent epidermal localization. Figure S6: H&E histology images of 90° MN insertion. Figure S7: H&E histology images of 45° MN insertion. Figure S8: H&E histology images of 20° MN insertion. Table S1: Percent epidermal localization for each insertion angle.
