# Supplementary material for: Angled Insertion of Microneedles for Targeted Antigen Delivery to the Epidermis
Source: Pharmaceutics. 2022 Feb 1;14(2):347. doi: 10.3390/pharmaceutics14020347 (PMC8874562; doi:10.3390/pharmaceutics14020347)
Supplement: Supplementary file 1 [file pharmaceutics-14-00347-s001.zip › pharmaceutics-1555612-supplementary.pdf]

pharmaceutics

Supplementary Materials: Angled Insertion of Microneedles for Targeted Antigen Delivery to the Epidermis

Rohan Murty, Abishek Sankaranarayanan, Isabella I. Bowland, Juan Mena-Lapaix and Mark R. Prausnit

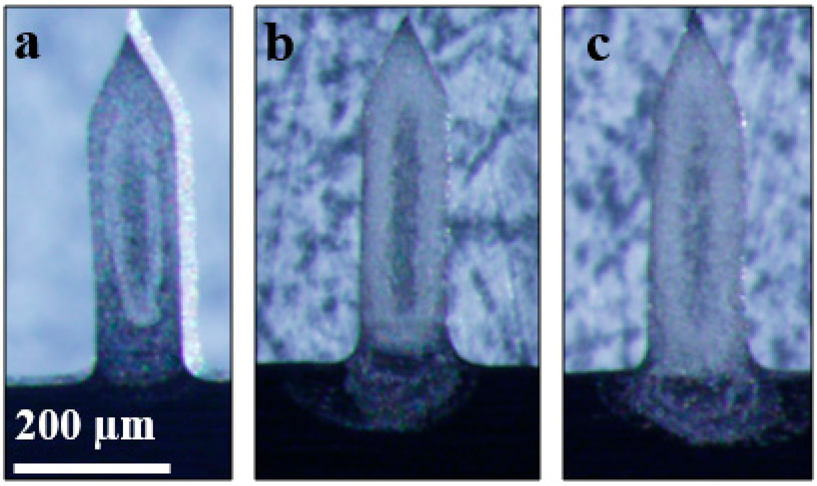

Figure S1. Representative images of MNs with coatings past the base of MNs. MNs measuring were coated using a formulation containing 1% (*w/v*) CMC, 0.5% (*w/v*) Pluronic F-68, 2% (*w/v*), and peanut antigen using (**a**) 5, (**b**) 10 or (**c**) 15 dip-coating cycles.

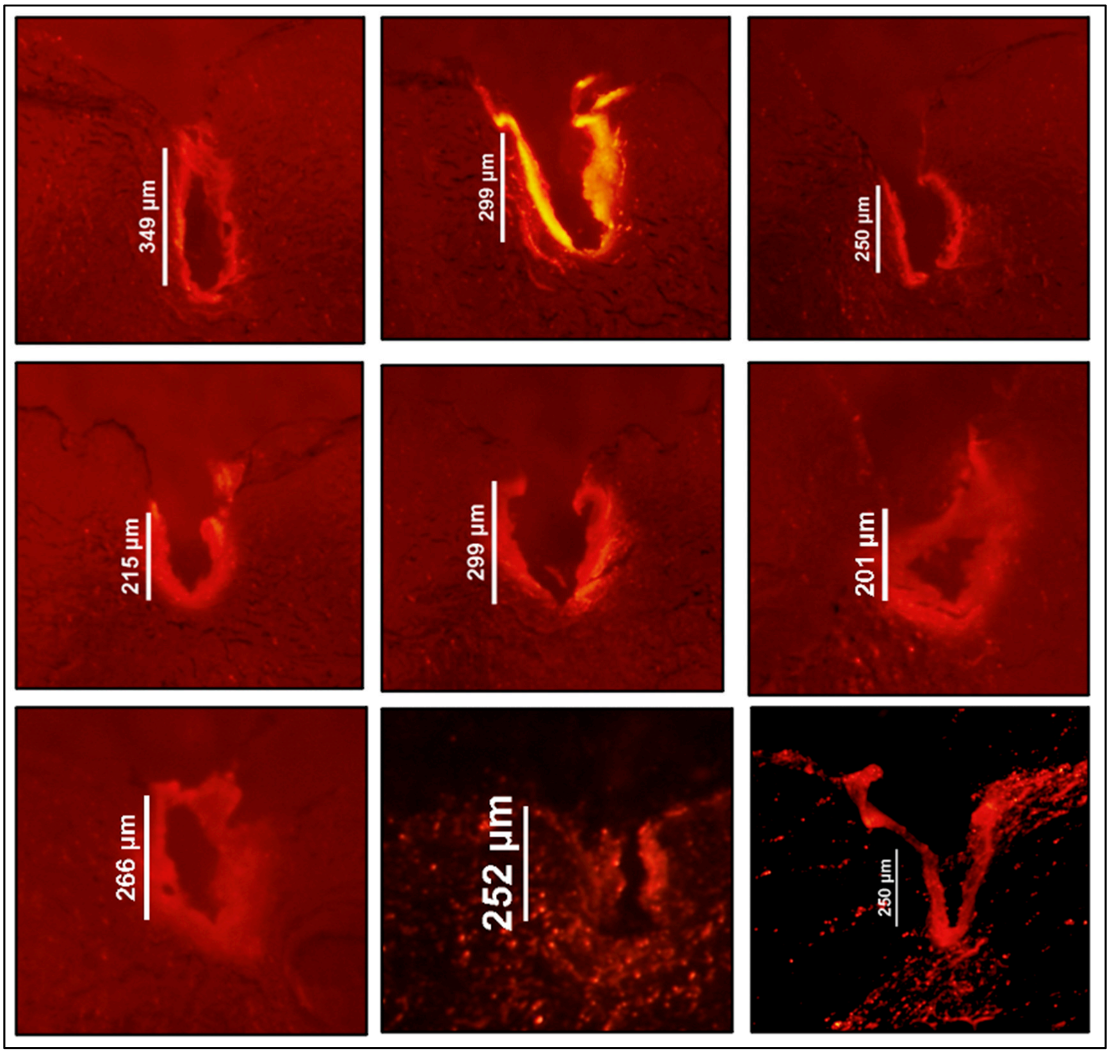

Figure S2. Images of histological skin sections after insertion of MNs coated with red-fluorescent sulforhodamine dye at an angle of 90° used to generated data presented in Figure 4.

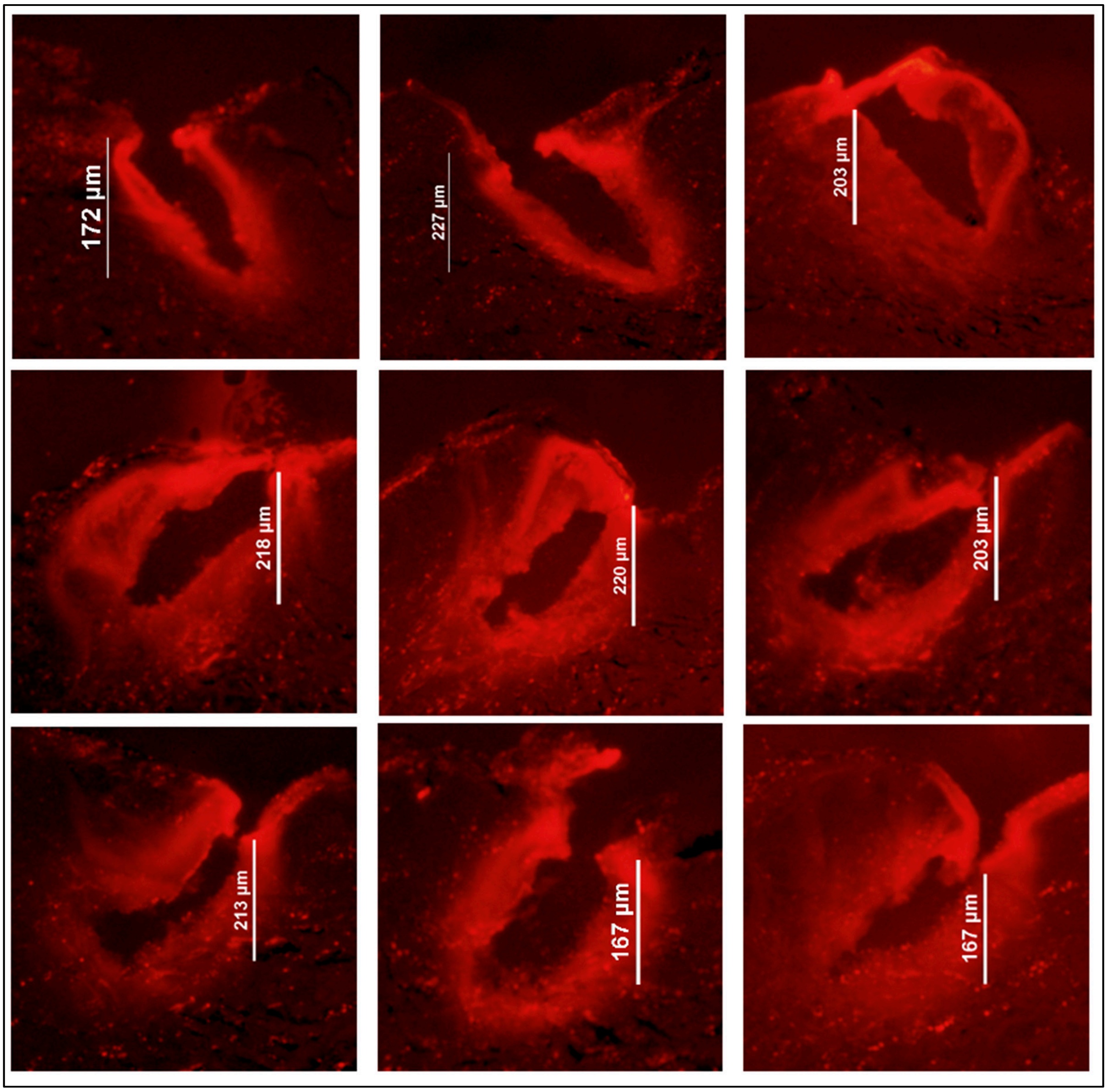

Figure S3. Images of histological skin sections after insertion of MNs coated with red-fluorescent sulforhodamine dye at an angle of 45° used to generated data presented in Figure 4.

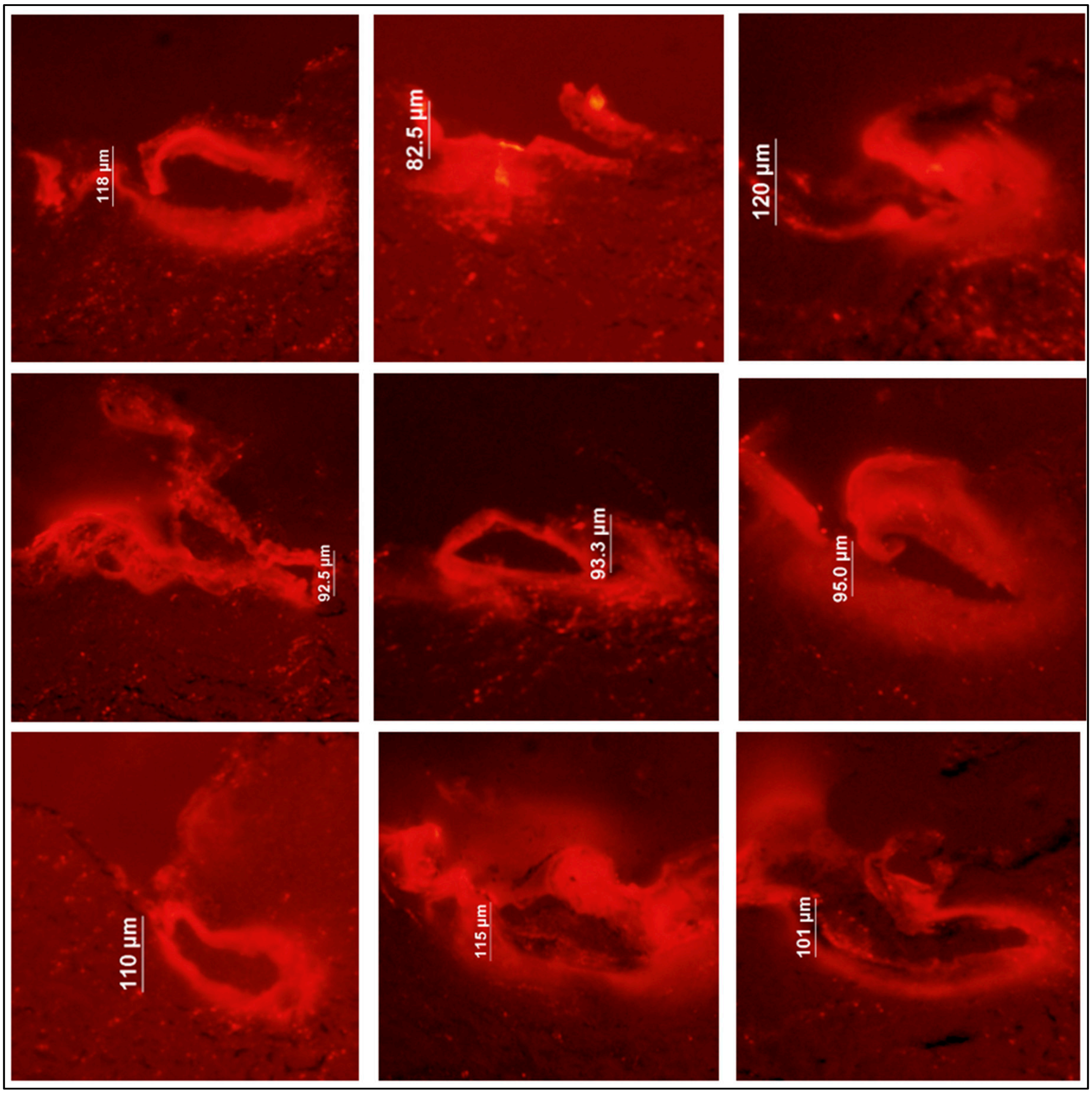

Figure S4. Images of histological skin sections after insertion of MNs coated with red-fluorescent sulforhodamine dye at an angle of 20° used to generated data presented in Figure 4.

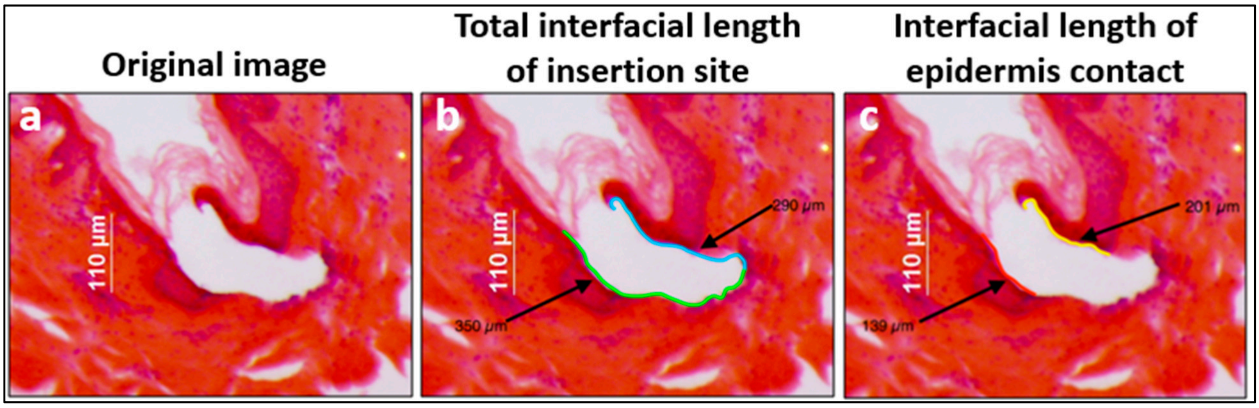

Figure 5. Representative H&E-stained images of 250 µm-long MNs inserted at 20° illustrating the method to calculate epidermis percent localization: (**a**) original insertion site image; (**b**) total interfacial length of the insertion site (640 µm); (**c**) interfacial epidermis contact length of the insertion site (340 µm).

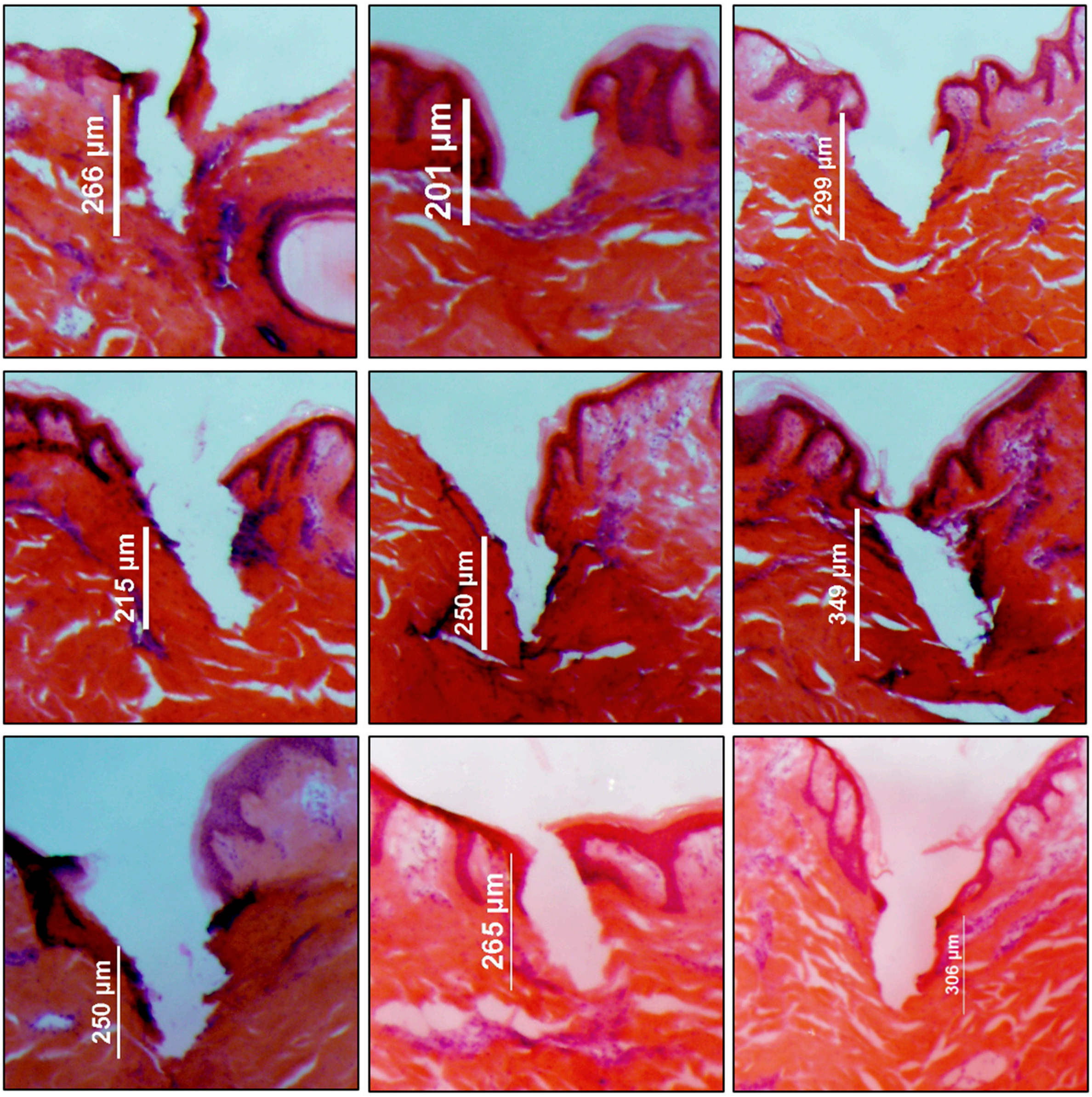

Figure S6. Images of histological skin sections stained with H&E after insertion of MNs at an angle of 90° used to generated data presented in Figure 5.

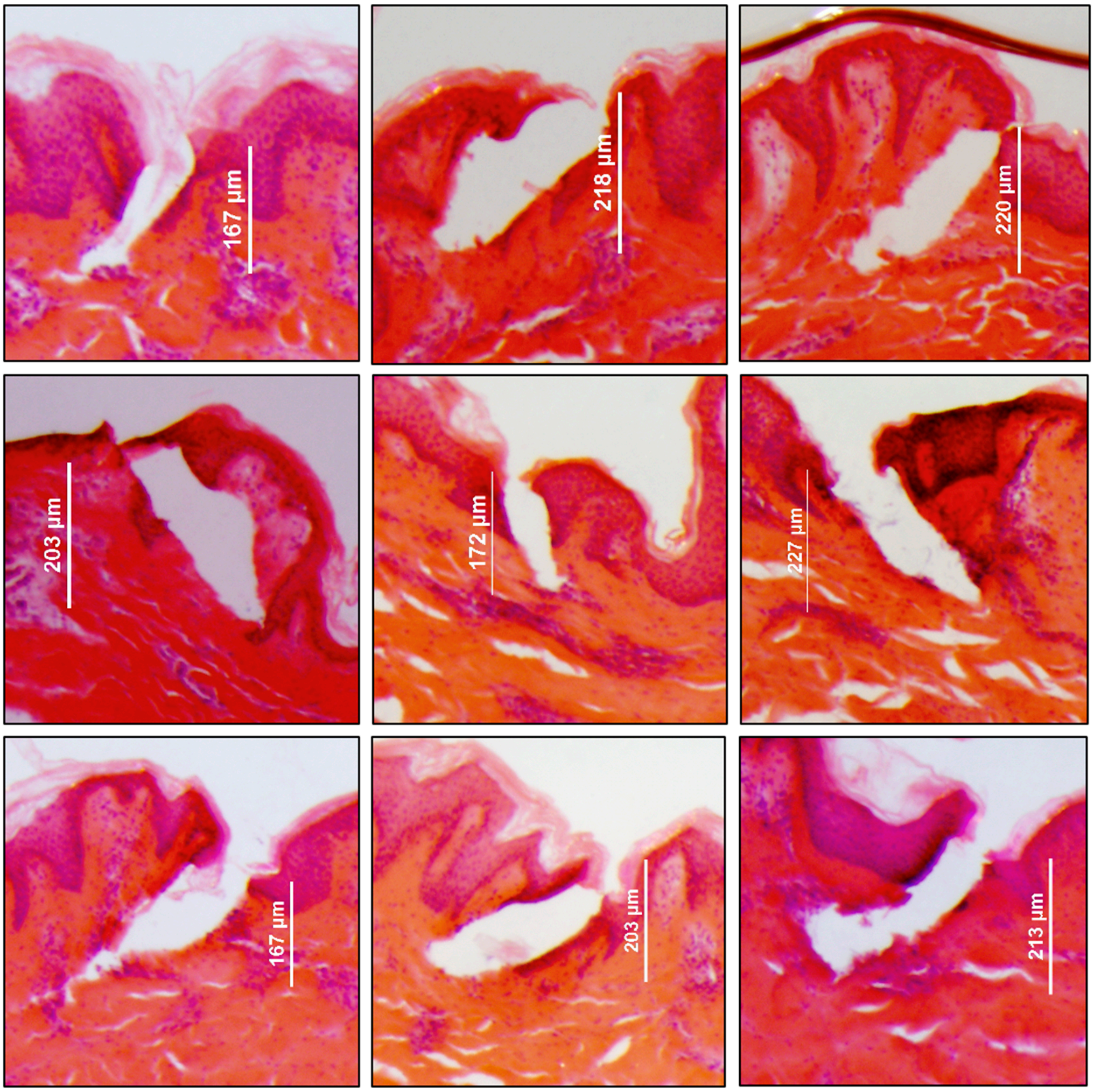

Figure S7. Images of histological skin sections stained with H&E after insertion of MNs at an angle of 45° used to generated data presented in Figure 5.

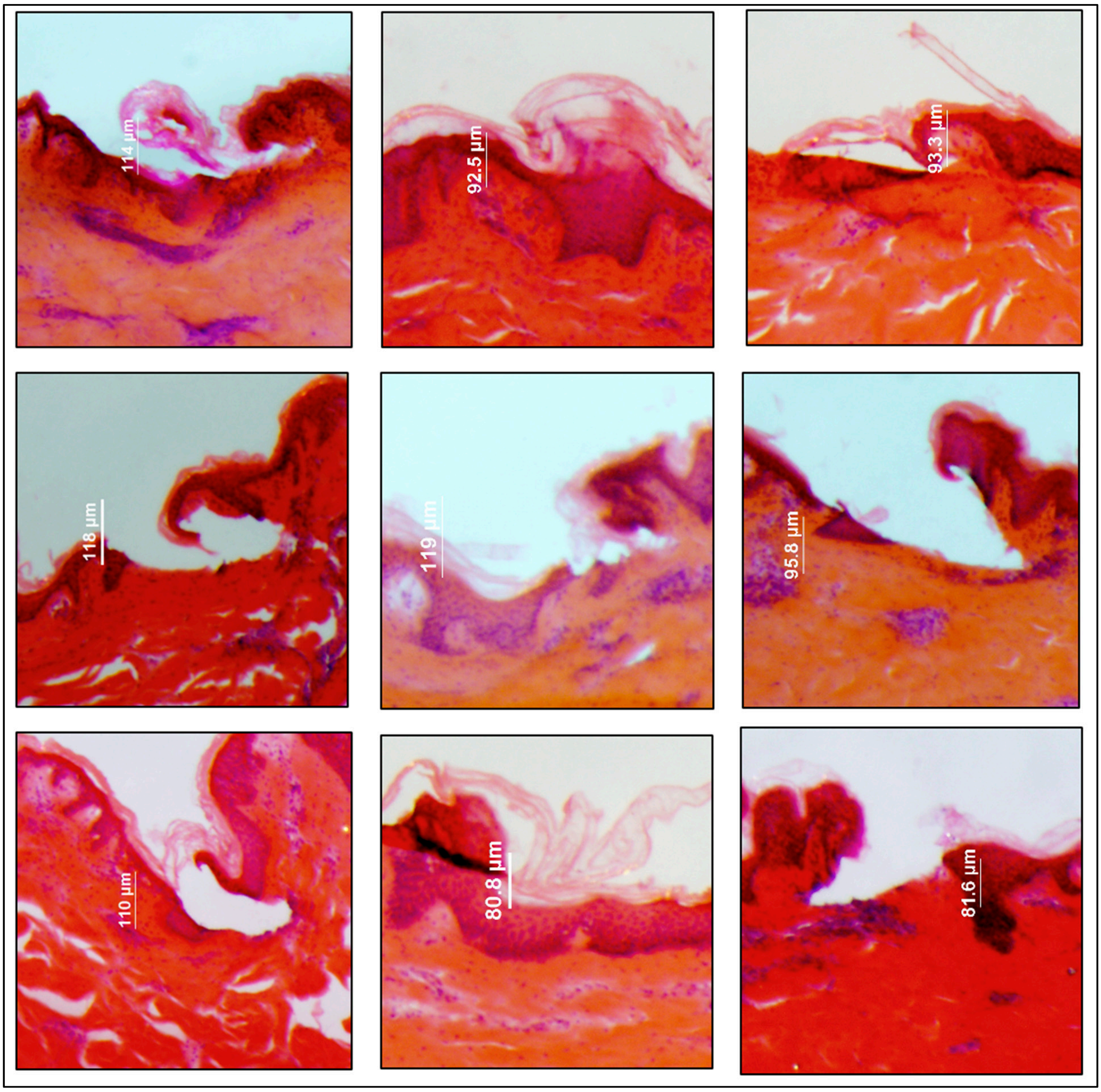

Figure S8. Images of histological skin sections stained with H&E after insertion of MNs at an angle of 20° used to generated data presented in Figure 5.

Table S1. Percent epidermal localization and associated statistics for each of the three insertion angles studied.

**Percent Epidermal Localization**				
		**Angle of Insertion**		
		**90°**	**45°**	**20°**
**Replicate**	1	16.8%	52.6%	100.0%
	2	39.1%	46.9%	100.0%
	3	25.0%	18.9%	72.0%
	4	40.9%	34.9%	47.5%
	5	11.9%	26.5%	83.4%
	6	8.7%	44.0%	61.0%
	7	18.7%	61.4%	52.9%
	8	45.5%	44.0%	67.3%
	9	18.4%	53.8%	43.0%
**Statistics**	**Mean**	**25.0%**	**42.6%**	**69.7%**
	**StDev**	**13.5%**	**13.6%**	**21.2%**
